# Clinical outcomes and patterns of failure of head and neck mucosal melanoma treated with multiple treatment modalities

**DOI:** 10.1186/s13014-021-01860-z

**Published:** 2021-07-28

**Authors:** Qing-Qing Xu, Yan-Zhen Lai, Zi-Lu Huang, Zi-Yi Zeng, Ya-Ni Zhang, Rui-Yao Ou, Wen-Min Wu, Lei Chen, Li-Xia Lu

**Affiliations:** 1 Department of Radiation Oncology, Sun Yat-Sen University Cancer Center, State Key Laboratory of Oncology in South China, Collaborative Innovation Center for Cancer Medicine,, Guangdong Key Laboratory of Nasopharyngeal Carcinoma Diagnosis and Therapy, 651 Dongfeng Road East, Guangzhou, 510060 China; 2 Heyuan People’s Hospital, Heyuan, China

**Keywords:** Head and neck mucosal melanoma, Treatment modalities, Clinical outcome, Prognosis, Immunologic/targeted therapy

## Abstract

**Objectives:**

The study aims to analyze the clinical characteristics of head and neck mucosal melanoma (MMHN) and the effects of multiple treatment modalities on distant metastasis, recurrence and survival rates to provide a reference for the individualized treatment of MMHN.

**Methods:**

We retrospectively reviewed 262 patients with stage III–IVb MMHN treated from March 1986 to November 2018 at our cancer center.

**Results:**

The median follow-up time was 34.0 months (range 1–262 months). The 5-year overall survival (OS), distant metastasis-free survival (DMFS) and disease-free survival (DFS) probabilities were 37.7%, 30.2%, and 20.3%, respectively. The 5-year OS rates for patients with stage III, stage IVA, and stage IVB MMHN were 67.0%, 24.1% and 8.3%, respectively (*P* < 0.001). A total of 246 (93.9%) patients received surgery, 149 (56.9%) patients received chemotherapy, and 69 (26.3%) patients received immunologic/targeted therapy. A total of 106 (40.5%) patients were treated with radiotherapy: 9 were treated with preoperative radiotherapy, 93 were treated with postoperative radiotherapy, and 4 were treated with radiotherapy alone. In the multivariate Cox regression analysis, primary tumor site, T stage, and immunologic/targeted therapy were independent factors for OS (all *P* < 0.05). Irradiation technique, T stage, and N stage were independent prognostic factors for DMFS (all *P* < 0.05). T stage, N stage, and surgery were independent prognostic factors for DFS (all *P* < 0.05). Distant metastasis was observed in 107 of 262 patients (40.8%), followed by local [74 (28.2%)] and regional [52 (19.8%)] recurrence.

**Conclusions:**

The main reason for treatment failure in MMHN is distant metastasis. Immunologic/targeted therapy and surgery are recommended to improve the survival of MMHN. The American Joint Committee on Cancer (AJCC) 8th edition staging system for MMHN does stage this disease effectively.

**Supplementary Information:**

The online version contains supplementary material available at 10.1186/s13014-021-01860-z.

## Introduction

Mucosal melanoma (MM) is a malignant neoplasm that accounts for approximately 0.8–3.7% of all melanomas [1, 2]. MM is the second most common subtype in China accounting for 20%-25% of all melanomas [3, 4], while is extremely rare in western countries accounting for approximately 1.3% of all melanomas [1, 5]. The head and neck region has the highest incidence of MM, accounting for 55% of all cases [1]. The most common sites of head and neck mucosal melanoma (MMHN) are the sinonasal cavity and oral cavity [6, 7]. It is less commonly found in the nasopharynx, oropharynx, eyelids, and larynx [8, 9]. MMHN is more common in China than in Western countries; previous studies reporting MMHN from Western countries were based on small sample sizes [10, 11].

MMHN has a poor prognosis, with a 5-year overall survival (OS) rate of less than 30% [7, 8]. Compared to cutaneous melanoma, MMHN is more aggressive, with lower 3- and 5-year survival rates and higher distant metastasis and local recurrence rates. Because of the occult primary site and lack of characteristic clinical manifestations, MMHN is often confused with nasal benign obstruction. Most patients are at an advanced stage at diagnosis. The TNM stage of the American Joint Committee on Cancer (AJCC) is distinguished from the traditional tumor stage, with only stages T3 and T4, highlighting high-grade malignant neoplasms [12]. According to the National Comprehensive Cancer Network (NCCN) guidelines (2019), surgical treatment of early lesions is still preferentially recommended [13]. However, consensus for the standard treatment regimen of MMHN has not been reached, and the efficacy of subsequent adjuvant therapy needs to be clarified. Although integrated treatment modalities have achieved rapid progress, the local control and long-term survival of MMHN are still unsatisfactory and have not improved in recent years [14]. On account of its rarity, relevant literature is mostly based on small sample analysis, which still calls for further investigation with a larger sample size.

Based on these considerations, we conducted a retrospective study to summarize the clinical characteristics of MMHN and investigate related risk factors for the survival outcomes of MMHN to explore the optimal treatments. To our knowledge, our study included the largest sample size of MMHN in a single center.

## Materials and methods

### Patients

In total, 262 previously untreated patients with biopsy-proven stage III-IVb MMHN were retrospectively included between March 1986 and November 2018. The patients were restaged based on clinical documents, surgical records and imaging findings according to the 8th edition AJCC staging system for MMHN [15], which determined stages T3 to T4b based on whether disease was confined to the mucosa or penetrated deeper tissues. The exclusion criteria were as follows: (1) evidence of distant metastasis before treatment, secondary malignancy, or both; (2) pregnancy or lactation; and (3) incomplete previous medical history and treatment process, relevant auxiliary examinations, pathological conditions and follow-up information. The ethics committee of our hospital approved our study protocol.

### Treatment

The treatment modalities were mainly divided into four groups: surgery alone (n = 83), surgery combined with radiotherapy (n = 26), surgery combined with chemotherapy (n = 61) and surgery combined with chemoradiotherapy (n = 76). Sixteen patients did not undergo surgery (three refused surgery, and thirteen had no surgical indications). Instead, four of them received radiotherapy alone, and twelve of them were treated with palliative chemotherapy. At present, it is widely believed that surgery is the preferred treatment for patients with MMHN at early age. According to the NCCN guidelines (2019) [13], surgery is the preferred treatment for stage T3 and T4a lesions, and external expansion of 1.5–2.0 cm at the surgical margin is recommended. Surgery (local resection or extended resection) was performed on 246 of 262 patients, of whom 107 underwent more than one surgery. For locally advanced and unresectable MMHN, radiotherapy is an alternative and was performed in 106 patients: 9 patients with preoperative radiotherapy, 93 patients with postoperative radiotherapy, and 4 patients with radiotherapy alone. Radiotherapy alone is often used as a palliative treatment for unresectable stage T4b disease. Twenty-two patients received 2D radiotherapy (2DRT), and 84 patients received intensity-modulated radiotherapy (IMRT). Details in radiotherapy target delineation were shown in Additional file 1 (page 4). A total of 149 patients received chemotherapy including dacarbazine, cisplatin, paclitaxel, and vindesine. Immunologic/targeted therapy mainly including IL-2, IFN-a-2b, and PD-1 antibodies was given to 69 patients.

### Follow-up and endpoints

The endpoints were clinical outcomes, including OS, distant metastasis-free survival (DMFS), disease-free survival (DFS), and locoregional relapse-free survival (LRRFS). Beginning from day 1 of treatment, OS was defined as the time to the date of death or patient censoring, whichever occurred first; DMFS was defined as the time to distant metastasis, death, or patient censoring, whichever occurred first; DFS was defined as the time to failure, death from any cause, or patient censoring, whichever occurred first; and LRRFS was defined as the time to local/regional relapse, death, or patient censoring, whichever occurred first. The patients were evaluated once every 3 months within the first 3 years of follow-up and every 6 months thereafter until death. The median follow-up time was 34 months (range: 1–262 months). After treatment, the patients were evaluated every 3 months during the first year and every 6 months thereafter.

### Statistical analysis

We used Kaplan–Meier survival curves to analyze the time-to-event endpoints (survival outcomes) of different patient subsets and used the log-rank test to assess the differences among them. The Cox proportional hazards model was used to perform multivariate analyses, which included the following variables: sex, age (< 50 years vs. ≥ 50 years), T classification (T3 vs. T4a vs. T4b), and N classification (N0 vs. N1). tumor stage, tumor characteristics, and treatment modalities. Statistical analyses were performed using SPSS version 22.0 (IBM Corporation, Armonk, NY, USA) and Stata software, version 14.2 (StataCorp). P values < 0.05 was considered significant. Hazard ratios (HRs) and 95% confidence intervals (CIs) were used as summary statistics for time-to-event data.

## Results

### Patient characteristics

The baseline characteristics are listed in Table 1. There were 262 patients: 101 females (38.5%) and 161 males (61.5%). The onset age ranged from 19 to 87 years, and the median age was 57 years. The primary tumor sites were as follows: nasal cavity in 139 (53%) patients, paranasal sinus in 22 (8.4%) patients, and oral cavity in 67 (25.6%) patients. A total of 34 (13%) patients had tumors in other sites such as the nasopharynx, oropharynx, eyelids, and larynx.

**Table 1:** Demographic and clinical characteristics of the patients at baseline

Characteristic	No. (%)
*Sex*	
Male	161 (61.5)
Female	101(38.5)
*Age*	
Median	57
Range	(19, 87)
*Smoke*	
Yes	73 (27.9)
No	189 (72.1)
*Primary tumour site*	
Nasal cavity	139 (53)
Paranasal sinus	22 (8.4)
Oral cavity	67 (25.6)
Others	34 (13)
*Tumor classification*	
T3	114 (43.5)
T4a	136 (51.9)
T4b	12 (4.6)
*Node classification*	
N0	195(74.4)
N1	67 (25.6)
*Overall stage*	
III	85 (32.4)
IVa	165 (63)
IVb	12 (4.6)
*Reoperation*	
Yes	107 (43.5)
No	139 (56.5)
*Irradiation technique*	
2DRT	22 (18.9)
IMRT	84 (81.3)
*Surgery*	
Yes	246 (93.9)
No	16 (6.1)
*Radiotherapy*	
Yes	106 (40.5)
No	156 (59.5)
*Chemotherapy*	
Yes	149 (56.9)
No	113 (43.1)
*Immunologic/targeted therapy*	
Yes	69 (26.3)
No	193 (73.7)
*Treatment modality*	
Surgery alone	83 (33.7)
Surgery + RT	26 (10.6)
Surgery + CT	61 (24.8)
Surgery + RT + CT	76 (30.9)

### Overall failure and survival

The median follow-up time for MMHN patients was 34 months (range, 1–262 months). During follow-up, primary recurrence included local recurrence in 74 (28.2%) patients and regional recurrence in 52 (19.8%) patients. Distant metastasis was observed in 107 patients (40.8%). The lungs (19.5%), the liver (16%), bones (12.6%), and the brain (6.9%) were several sites with a high incidence of distant metastases. All distant metastases, local relapses, and regional recurrences occurred in 59 (22.5%) patients.

**Fig. 1:**
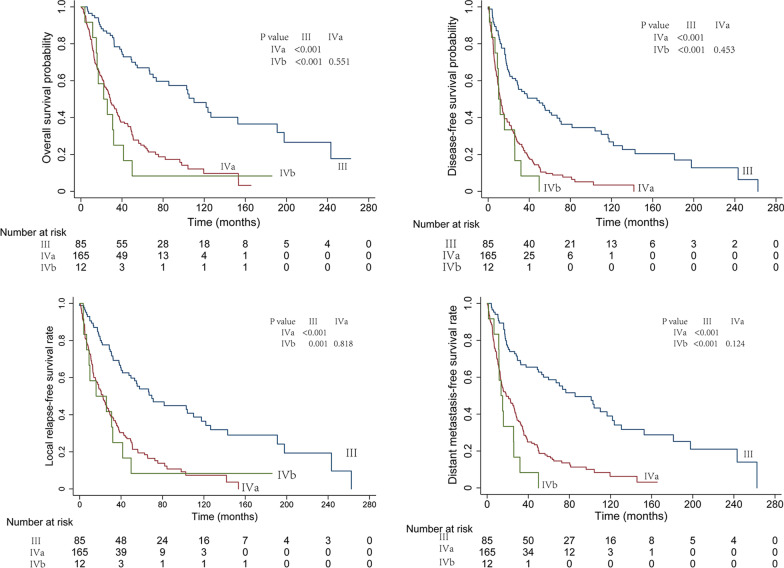
Survival curves according to the 8th AJCC stage III, IVa, and IVb

The 5-year OS, DMFS, DFS, and LRRFS rates were 37.7%, 30.2%, 20.3%, and 21.0%, respectively. Among patients with stage III, IVa, and IVb disease, the 3-year OS rates were 77.0%, 41.3% and 25.0%, respectively (*P* < 0.001); DFS rates were 52.9%, 20.9% and 8.3%, respectively (*P* < 0.001); LRRFS rates were 55.7%, 29.0% and 16.7%, respectively (*P* < 0.001); DMFS rates were 65.5%, 28,5% and 8.3%, respectively (*P* < 0.001) (Fig. 1).

### Clinical outcomes

**Fig. 2:**
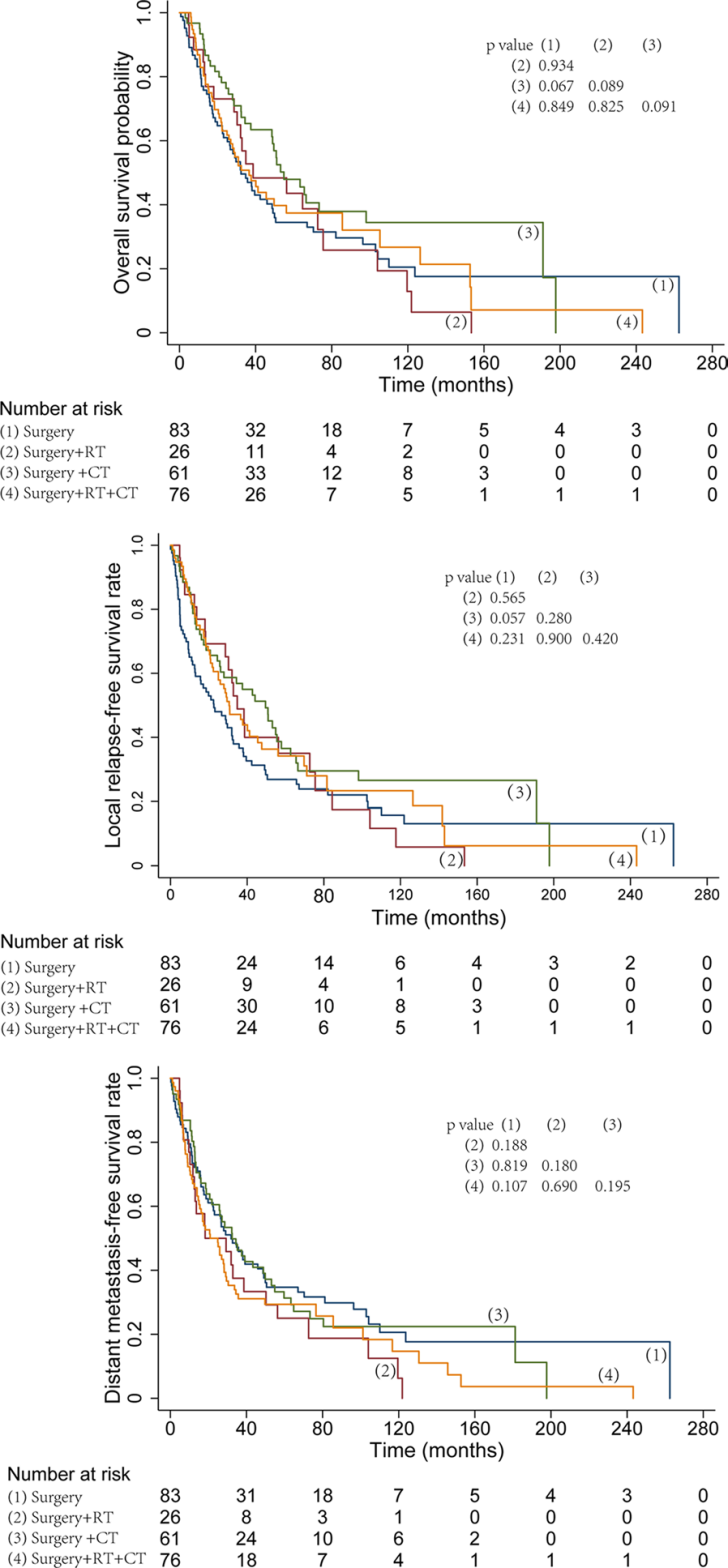
Survival curves according to different treatment modalities

**Fig. 3:**
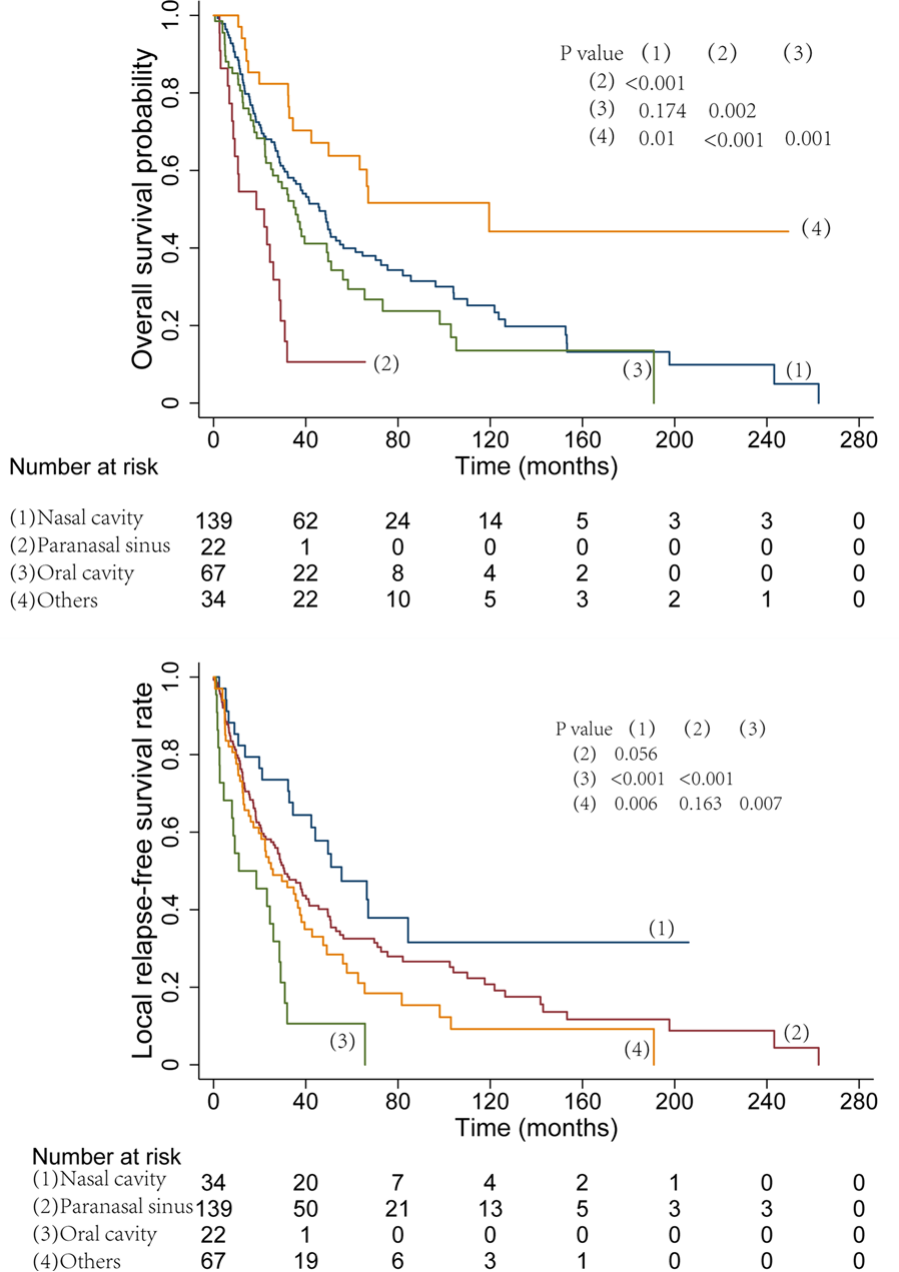
Survival curves according to different primary tumor locations

**Fig. 4:**
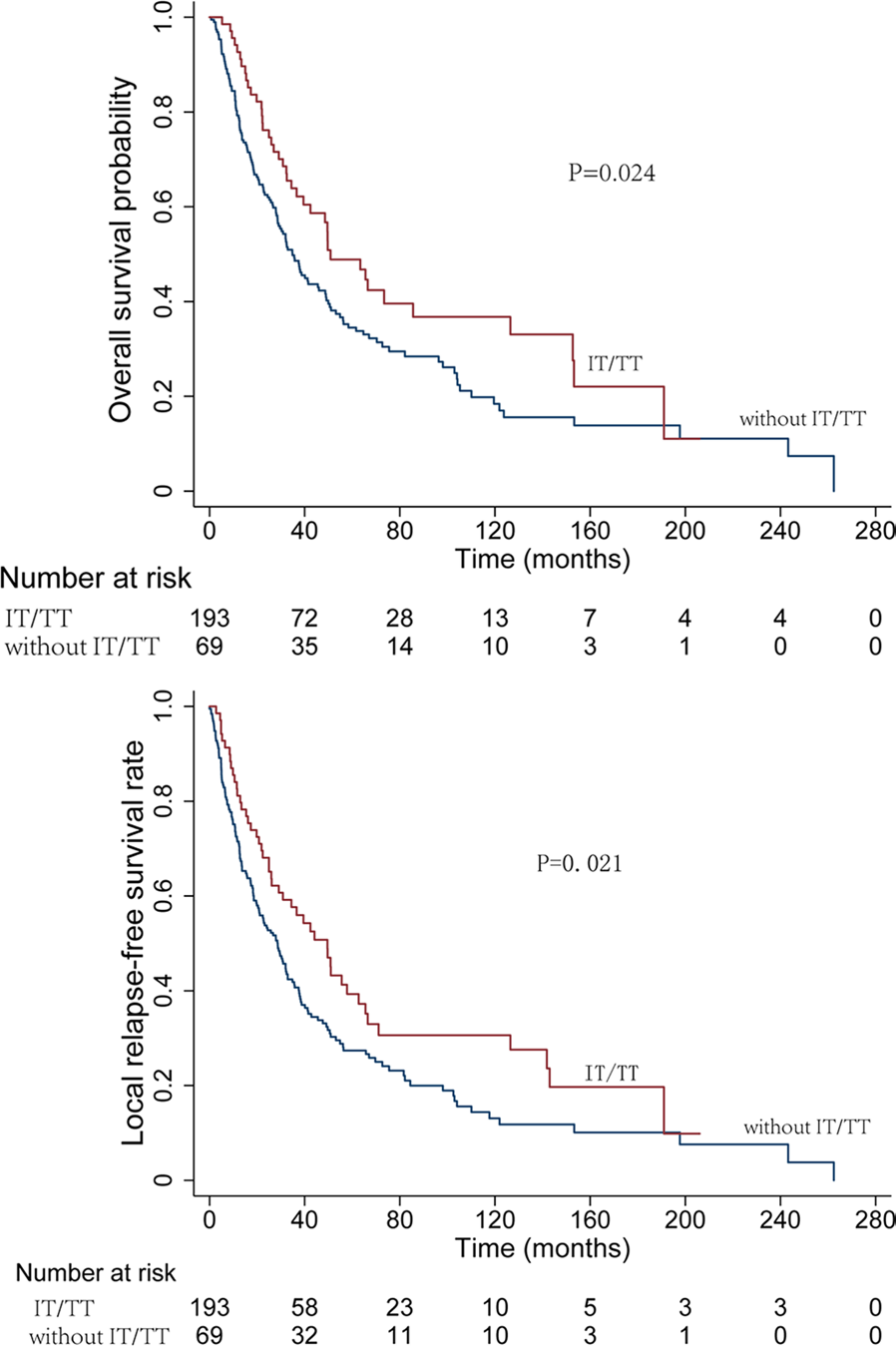
Survival curves of patients with or without immunologic/targeted therapy

By different treatment modalities, the corresponding 5-year OS, LRFS and DMFS rates were as follows: surgery alone, 33.0%, 25.3%, and 33.2%; surgery combined with radiotherapy, 38.7%, 35.0%, and 25.0%; surgery combined with chemotherapy, 45.5%, 34.2%, and 31.3%; and surgery combined with chemoradiotherapy, 37.4%, 34.2%, and 29.4% (Fig. 2). According to different primary tumor locations, the corresponding 3-year OS, and LRFS were as follows: nasal cavity, 70.3%, and 64.4%; paranasal sinus, 56.5%, and 46.9%; oral cavity, 10.6%, and 10.6%; and other sites, 46.7%, and 40.5% (Fig. 3). There were no obvious differences in the OS rate (HR = 0.733, 95% CI 0.349–1.540, *P* = 0.413) or local control rate (HR = 0.586, 95% CI 0.172–1.992, *P* = 0.392) between the preoperative radiotherapy group and the postoperative radiotherapy group. In addition, there were no obvious differences in the local control rate (HR = 0.726, 95% CI 0.438–1.202, *P* = 0.213) or DMFS rate (HR = 1.270, 95% CI 0.949–1.701, *P* = 0.108) between the surgery alone group and the surgery plus postoperative radiotherapy group. The 5-year OS and LRFS of patients treated with and without immunologic/targeted therapy were analyzed, and the corresponding rates are as follows: with immunologic/targeted therapy, 62.2% and 56.0%; without immunologic/targeted therapy, 48.6% and 40.7% (Fig. 4).

### Univariate and multivariate analysis

The results of univariate and multivariate analyses of prognostic factors for OS, DMFS, DFS, and LRRFS are listed in Tables 2 and 3, respectively. Primary tumor location, tumor stage, and immunologic/targeted therapy were independent factors affecting OS prognosis (all *P* < 0.05). Irradiation technique (2DRT and IMRT), tumor stage, and node classification were independent prognostic factors for DMFS (all *P* < 0.05). Tumor stage, node classification, and surgery were independent prognostic factors for DFS (all *P* < 0.05).

**Table 2:** Summary of univariate analysis of prognostic factors

Endpoint	Variable	HR	95% CI for HR	*P* value^a^
OS	Primary tumour site			**0.029**
	Nasal cavity	1.749	(1.096, 2.791)	
	Paranasal sinus	3.438	(2.974, 6.525)	
	Oral cavity	2.032	(1.222, 3.378)	
	Others(reference)	1		
	Reoperation	0.653	(0.477, 0.894)	**0.008**
	T classification			** < 0.001**
	T3(reference)	1		
	T4a	3.263	(2.336, 4.560)	
	T4b	3.407	(1.776, 6.536)	
	Node classification	1.641	(1.181, 2.281)	**0.003**
	Immunologic/targeted therapy	0.671	(0.473, 0.952)	**0.025**
DMFS	Primary tumour site			**0.002**
	Nasal cavity	1.749	(1.096, 2.791)	
	Paranasal sinus	3.438	(1.811, 6.525)	
	Oral cavity	2.032	(1.222, 3.378)	
	Others(reference)	1		
	Reoperation	0.721	(0.539, 0.964)	**0.027**
	Irradiation technique	1.723	(0.987, 3.010)	0.056
	T classification			** < 0.001**
	T3(reference)	1		
	T4a	2.897	(2.121, 3.957)	
	T4b	4.282	(2.279, 8.044)	
	N classification	1.725	(1.267, 2.349)	**0.001**
DFS	Primary tumour site			**0.001**
	Nasal cavity	1.584	(1.020, 2.459)	
	Paranasal sinus	2.880	(1.576, 5.262)	
	Oral cavity	2.260	(1.405, 3.636)	
	Others(reference)	1		
	Irradiation technique	1.943	(1.125, 3.358)	**0.017**
	T classification			** < 0.001**
	T3(reference)	1		
	T4a	2.363	(1.772, 3.152)	
	T4b	2.848	(1.539, 5.270)	
	Node classification	1.775	(1.315, 2.396)	** < 0.001**
	Smoke	0.754	(0.559, 1.015)	0.063
	Surgery	0.592	(0.355, 0.989)	**0.045**
LRRFS	Primary tumour site			** < 0.001**
	Nasal cavity	1.878	(1.177, 2.995)	
	Paranasal sinus	3.848	(2.068, 7.161)	
	Oral cavity	2.602	(1.579, 4.287)	
	Others(reference)	1		
	Irradiation technique	1.610	(0.935, 2.770)	0.086
	T classification			** < 0.001**
	T3(reference)	1		
	T4a	2.148	(1.610, 2.864)	
	T4b	2.705	(1.463, 5.003)	
	Node classification	1.712	(1.263, 2.320)	**0.001**
	Surgery	0.581	(0.348, 0.971)	**0.038**

^a^Statistically significant results are shown in bold. *P values < 0.05 are highlighted in bold

OS, overall survival; DMFS, distant metastasis-free survival; DFS: disease-free survival probability; LRRFS, local regional relapse free survival; HR, hazard ratio; CI, confidence interval

**Table 3:** Summary of multivariate analysis of prognostic factors

Endpoint	Variable	HR	95% CI for HR	*P* value^a^
OS	Primary tumour site			**0.003**
	Nasal cavity	1.467	(0.842, 2.555)	
	Paranasal sinus	2.974	(1.578, 6.350)	
	Oral cavity	2.404	(1.539, 4.986)	
	Others(reference)	1		
	Reoperation	0.733	(0.530, 1.015)	0.061
	T classification			**< 0.001**
	T3(reference)	1		
	T4a	3.073	(2.146, 4.401)	
	T4b	3.817	(1.854, 7.857)	
	Immunologic/targeted therapy	0.638	(0.435, 0.934)	**0.021**
DMFS	Irradiation technique	2.145	(1.158, 3.975)	**0.015**
	T classification			**< 0.001**
	T3(reference)	1		
	T4a	2.524	(1.560, 4.085)	
	T4b	4.025	(1.348, 12.018)	
	N classification	1.808	(1.037, 3.154)	**0.037**
DFS	T classification			**0.001**
	T3(reference)	1		
	T4a	2.315	(1.466, 3.658)	
	T4b	3.016	(1.093, 8.326)	
	Node classification	2.298	(1.338, 3.948)	**0.003**
	Irradiation technique	1.767	(0.978, 3.190)	0.059
	Smoking	0.607	(0.355, 1.040)	0.069
	Surgery	0.311	(0.104, 0.929)	**0.036**
LRRFS	T classification			**0.015**
	T3(reference)	1		
	T4a	1.798	(1.131, 2.858)	
	T4b	2.978	(1.125, 7.878)	
	Node classification	1.916	(1.097, 3.345)	**0.022**
	Irradiation technique	1.660	(0.941, 2.949)	0.08

^a^Statistically significant results are shown in bold. *P values < 0.05 are highlighted in bold

OS, overall survival; DMFS, distant metastasis-free survival; DFS: disease-free survival probability; LRRFS, local regional relapse free survival; HR, hazard ratio; CI, confidence interval

#### Treatment-related adverse events

Acute and late toxicities were defined as events occurring within 90 days, and 90 days after radiotherapy initiation, respectively. The results were listed in Table 4. Most patients experienced acute grade 1–2 anemia (32.1%) and dermatitis (22.9%). Nausea (10.7%), vomiting (6.9%), mucositis (9.9%) and anemia (13.0%) were the most recorded grade 3–4 adverse events.

**Table 4:** Acute and late toxicities

Event	Grades 1–2	Grade 3–4
*Acute toxicities*		
Hematological		
Anemia	84 (32.1%)	14 (5.3%)
Thrombocytopenia	9 (3.4%)	2 (0.8%)
Leucopenia	23 (8.8%)	5 (1.9%)
Neutropenia	18 (6.9%)	6 (2.3%)
Nonhematological		
Dermatitis	60 (22.9%)	4 (1.5%)
Mucositis	42 (16.0%	8 (3.1%)
Dry mouth	16 (6.1%)	4 (1.5%)
Nausea	46 (17.6%)	9 (3.4%)
Vomiting	41 (15.6%)	0
Keratitis	2 (7.7%)	0
*Late toxicities*		
Hematological		
Anemia	34 (13.0%)	4 (1.5%)
Thrombocytopenia	2 (0.8%)	0
Leucopenia	13 (5.0%)	0
Neutropenia	11 (4.2%)	2 (0.8%)
Nonhematological		
Nausea	28 (10.7%)	5 (1.9%)
Vomiting	18 (6.9%)	2 (0.8%)
Mucositis	26 (9.9%)	4 (1.5%)
Nephrotoxicity	8 (3.1%)	1 (0.8%)
Hepatitis	7 (2.7%)	2 (0.8%)
Neurotoxicity	4 (1.5%)	0
Cervical fibrosis	2 (0.8%)	0

## Discussion

MMHN is a rare entity. We included 262 patients with MMHN, which to our knowledge constituted one of the largest single-institution cohorts of MMHN patients, to investigate the effect of multiple treatment modalities on MMHN. We found that immunologic/targeted therapy, irradiation technique, and surgery were independent factors for prognosis.

To date, there is no consensus on the treatment modalities for MMHN. The existing guidelines all regard surgery as the preferred method of treatment [13]. Meanwhile, routine adjuvant radiotherapy post-operation to improve the local control rate and the use of systemic treatment for advanced disease and disease progression are recommended. A meta-analysis including 12 retrospective studies (n = 1593) showed that surgery plus postoperative radiotherapy reduced the risk of local recurrence but did not reduce the risk of death or distant metastasis [16]. Similar results were found in several other studies [17–19]. A summary of several studies evaluating the effect of multiple treatment in MMHN was shown in the Additional file 1 (page 3) [6, 7, 20]. In our study, we found that surgery plus postoperative radiotherapy was not associated with the LRFS or DMFS rate. There may be several reasons for this. First, due to the low incidence of MMHN and the lack of a large-sample study, there was a large bias in case selection. Patients who had been chosen to receive postoperative radiotherapy tended to have wide lesion invasion, which may have offset some of the survival benefits of postoperative radiotherapy. Second, MMHN is a systemic disease that is very prone to invading blood vessels and lymphatic tissues and is very prone to developing distant metastasis due to its unique satellite and microsatellite foci. Although postoperative radiotherapy can achieve better local control, it cannot reduce the risk of distant metastasis. Therefore, increasing the intensity of local treatment to improve survival is not optimistic at this stage, and more clinical studies should seek treatment to reduce the risk of distant recurrence. Chemotherapy may be used in metastatic and unresectable patients. However, chemotherapy is mostly used in the treatment of cutaneous melanoma, and the therapeutic effect of chemotherapy on MMHN remains unknown. In recent years, immunologic/targeted therapy based on patients' genetic changes has been gradually applied to patients with mucosal melanoma, and our study also confirmed that the application of immunologic/targeted therapy had a beneficial impact on prognosis. Relevant studies have shown that the phenotypic changes related to MMHN include C-Kit, NRAS, and GNAQ [21–23], which are the targets of some targeted drugs, including vemurafenib, imatinib, and nilotinib [24]. Tumor-mediated immune tolerance is a focus at present. Nivolumab can act as an immune checkpoint inhibitor to block the suppression of activated T cells, thereby conducting immune surveillance of cancer cells. D 'Angelo et al. [25] reported that the overall response rates of MM with combination therapy were 37% (ipilimumab + nivolumab), 23% (nivolumab alone) and 8.3% (ipilimumab alone). These studies indicate that immunotherapy also plays an important role in the treatment of MM. Ferroptosis is a kind of iron-dependent cell death, and apoptosis is essentially caused by the accumulation of lipid peroxides. A recent study revealed that ferroptosis is associated with immunotherapy [26]. Researchers have found that when immunotherapy enhances T cell activity, it can increase the level of lipid-specific oxidation in tumor cells and even cause tumor cell death. Studies based on mice and cancer cells have found that increasing ferroptosis makes immunotherapy more effective, which plays a promising role in MM treatment.

With regard to other prognostic factors, we found similar results as those of previous studies. Patients with MMHN who were ≤ 57 years old had a better 5-year OS rate (42.1% vs. 34.2%). We also identified an obvious difference in the OS rate with regard to different primary tumor locations. In contrast with primary tumors located in the oral and nasal cavities, primary tumors in the paranasal sinus had the worst prognosis, which was in accordance with the findings of several previous studies [11, 27]. In this paper, the 8th AJCC staging system for MMHN was applied, and T stage, N stage and overall stage were associated with prognosis.

The main limitation of this study was that it was a retrospective study from a single center; therefore, further prospective multicenter studies are needed. Nevertheless, the study had one of the largest sample sizes (n = 262) of MMHN in a single institution. Moreover, approximately 20% of the patients in the present paper were treated with conventional 2D or 3D radiation techniques rather than IMRT. This might be a confounding factor for the results since IMRT reduced toxicity, in particular oral mucositis, compared with conventional techniques for the treatment of head and neck cancer [28].

## Conclusions

In conclusion, MMHN is often accompanied by high recurrence and distant metastasis, as well as low 3- and 5-year survival rates. Radical surgery combined with postoperative adjuvant radiotherapy can improve the local control rate of early lesions, but systemic treatment is essential for patients with advanced stages and postoperative recurrence. When the disease progresses, applying immunologic/targeted therapy to improve the survival and prognosis of patients can be considered. The comprehensive treatment modality of MM is still under investigation, and a more effective comprehensive treatment regimen is needed. Thus, further prospective trials are warranted.

## Supplementary Information

**Additional file 1**. **Figure S1**. Survival outcomes between patients treated 10 years ago and within the last 10 years. **Table S1.** Distribution of the use of immunotherapy/targeted therapy in patients receiving surgery, radiotherapy, and chemotherapy, respectively.**Table S2.** Other studies evaluating the effect of multiple treatment in MMHN. **Dose and target delineation.**  The median radiotherapy dose was 58 Gy (range 30-70 Gy). Gross tumor volume (GTV) included the primary tumor and the enlarged lymph nodes, and GTV was expanded by 5-10 mm to generate the clinical tumor volume (CTV). For postoperative adjuvant radiotherapy, the CTV included the entire anatomical site where the tumors were located, and was expanded by 3-5 mm to generate the planning target volume (PTV). Cervical high-risk areas (including the number of metastatic lymph nodes ≥ 2, diameter ≥ 3 cm, extranodal lymph node invasion, and local recurrence after lymphatic dissection) were encompassed by the CTV.

## Data Availability

The datasets analyzed during the current study are available from the corresponding author on reasonable request.

## Abbreviations

AJCC: American Joint Committee on Cancer
CI: Confidence interval
HR: Hazard ratio
IMRT: Intensity modulated radiotherapy
OS: Overall survival
DMFS: Distant metastasis-free survival
DFS: Disease-free survival probability
LRRFS: Local regional relapse free survival
MMHN: Mucosal melanoma of head and neck

## References

[1] Chang AE, Karnell LH, Menck HR (1998). The National Cancer Data Base report on cutaneous and noncutaneous melanoma: a summary of 84,836 cases from the past decade. The American College of Surgeons Commission on Cancer and the American Cancer Society. Cancer.

[2] Manolidis S, Donald PJ (1997). Malignant mucosal melanoma of the head and neck: review of the literature and report of 14 patients. Cancer.

[3] Lian B, Cui CL, Zhou L, Song X, Zhang XS, Wu D (2017). The natural history and patterns of metastases from mucosal melanoma: an analysis of 706 prospectively-followed patients. Ann Oncol.

[4] Chi Z, Li S, Sheng X, Si L, Cui C, Han M (2011). Clinical presentation, histology, and prognoses of malignant melanoma in ethnic Chinese: a study of 522 consecutive cases. BMC Cancer.

[5] Yan X, Sheng X, Chi Z, Si L, Cui C, Kong Y (2021). Randomized phase II study of bevacizumab in combination with carboplatin plus paclitaxel in patients with previously unt. J Clin Oncol.

[6] Sun S, Huang X, Gao L, Zhang Y, Luo J, Zhang S (2017). Long-term treatment outcomes and prognosis of mucosal melanoma of the head and neck: 161 cases from a single institution. Oral Oncol.

[7] Schmidt MQ, David J, Yoshida EJ, Scher K, Mita A, Shiao SL (2017). Predictors of survival in head and neck mucosal melanoma. Oral Oncol.

[8] Jethanamest D, Vila PM, Sikora AG, Morris LGT (2011). Predictors of survival in mucosal melanoma of the head and neck. Ann Surg Oncol.

[9] Lee G, Baek C-H, Choi NY, Chung MK (2017). The prognostic role of the surgical approach and adjuvant therapy in operable mucosal melanoma of the head and neck. Clin Exp Otorhinolaryngol.

[10] Takagi M, Ishikawa G, Mori W (1974). Primary malignant melanoma of the oral cavity in Japan. With special reference to mucosal melanosis. Cancer.

[11] Cheng Y-F, Lai C-C, Ho C-Y, Shu C-H, Lin C-Z (2007). Toward a better understanding of sinonasal mucosal melanoma: clinical review of 23 cases. J Chin Med Assoc.

[12] Veronesi U, Boyle P, Goldhirsch A, Orecchia R, Viale G (2005). Breast cancer. Lancet (London, England).

[13] Treatment of ductal carcinoma in situ (2013). an uncertain harm-benefit balance. Prescrire Int.

[14] Ascierto PA, Accorona R, Botti G, Farina D, Fossati P, Gatta G (2017). Mucosal melanoma of the head and neck. Crit Rev Oncol Hematol.

[15] Dess RT, Suresh K, Zelefsky MJ, Freedland SJ, Mahal BA, Cooperberg MR (2020). Development and validation of a clinical prognostic stage group system for nonmetastatic prostate cancer using disease-specific mortality results from the international staging collaboration for cancer of the prostate. JAMA Oncol.

[16] Li W, Yu Y, Wang H, Yan A, Jiang X (2015). Evaluation of the prognostic impact of postoperative adjuvant radiotherapy on head and neck mucosal melanoma: a meta-analysis. BMC Cancer.

[17] Wushou A, Hou J, Zhao Y-J, Miao X-C (2015). Postoperative adjuvant radiotherapy improves loco-regional recurrence of head and neck mucosal melanoma. J Craniomaxillofac Surg.

[18] Jarrom D, Paleri V, Kerawala C, Roques T, Bhide S, Newman L (2017). Mucosal melanoma of the upper airways tract mucosal melanoma: a systematic review with meta-analyses of treatment. Head Neck.

[19] Yao J-J, Zhang F, Zhang G-S, Deng X-W, Zhang W-J, Lawrence WR (2018). Efficacy and safety of primary surgery with postoperative radiotherapy in head and neck mucosal melanoma: a single-arm Phase II study. Cancer Manag Res.

[20] Chen E, Wu J, Liu Z, Yang S, Wu J, Ouyang P (2021). Prognostic values of treatment modalities on head and neck mucosal melanomas in elderly patients: a population-based analysis. Ann Transl Med.

[21] Williams MD (2017). Update from the 4th edition of the world health organization classification of head and neck tumours: mucosal melanomas. Head Neck Pathol.

[22] Turri-Zanoni M, Medicina D, Lombardi D, Ungari M, Balzarini P, Rossini C (2013). Sinonasal mucosal melanoma: molecular profile and therapeutic implications from a series of 32 cases. Head Neck.

[23] Beadling C, Jacobson-Dunlop E, Hodi FS, Le C, Warrick A, Patterson J (2008). KIT gene mutations and copy number in melanoma subtypes. Clin Cancer Res.

[24] Beswick DM, Holsinger FC, Kaplan MJ, Fischbein NJ, Hara W, Colevas AD (2016). Design and rationale of a prospective, multi-institutional registry for patients with sinonasal malignancy. Laryngoscope.

[25] D'Angelo SP, Larkin J, Sosman JA, Lebbé C, Brady B, Neyns B (2017). Efficacy and safety of nivolumab alone or in combination with ipilimumab in patients with mucosal melanoma: a pooled analysis. J Clin Oncol.

[26] Wang W, Green M, Choi JE, Gijón M, Kennedy PD, Johnson JK (2019). CD8 T cells regulate tumour ferroptosis during cancer immunotherapy. Nature.

[27] Nakaya M, Mochiki M, Takeuchi S, Yuge T, Nakao K, Nakamura N (2004). Malignant melanoma of nasal cavity: report of 16 Japanese patients. Auris Nasus Larynx.

[28] Mazzola R, Ricchetti F, Fersino S, Fiorentino A, Giaj Levra N, Di Paola G (2016). Predictors of mucositis in oropharyngeal and oral cavity cancer in patients treated with volumetric modulated radiation treatment: A dose-volume analysis. Head Neck.

