# Supplementary material for: Clinical outcomes and patterns of failure of head and neck mucosal melanoma treated with multiple treatment modalities
Source: Radiat Oncol. 2021 Jul 28;16:138. doi: 10.1186/s13014-021-01860-z (PMC8317323; doi:10.1186/s13014-021-01860-z)
Supplement: Supplementary file 1 — Additional file 1. Figure S1. Survival outcomes between patients treated 10 years ago and within the last 10 years. Table S1. Distribution of the use of immunotherapy/targeted therapy in patients receiving surgery, radiotherapy, and chemotherapy, respectively.Table S2. Other studies evaluating the effect of multiple treatment in MMHN. Dose and target delineation. The median radiotherapy dose was 58 Gy (range 30-70 Gy). Gross tumor volume (GTV) included the primary tumor and the enlarged lymph nodes, and GTV was expanded by 5-10 mm to generate the clinical tumor volume (CTV). For postoperative adjuvant radiotherapy, the CTV included the entire anatomical site where the tumors were located, and was expanded by 3-5 mm to generate the planning target volume (PTV). Cervical high-risk areas (including the number of metastatic lymph nodes ≥ 2, diameter ≥ 3 cm, extranodal lymph node invasion, and local recurrence after lymphatic dissection) were encompassed by the CTV. [file 13014_2021_1860_MOESM1_ESM.docx]

**Figure S1.** Survival outcomes between patients treated 10 years ago and within the last

10 years

**
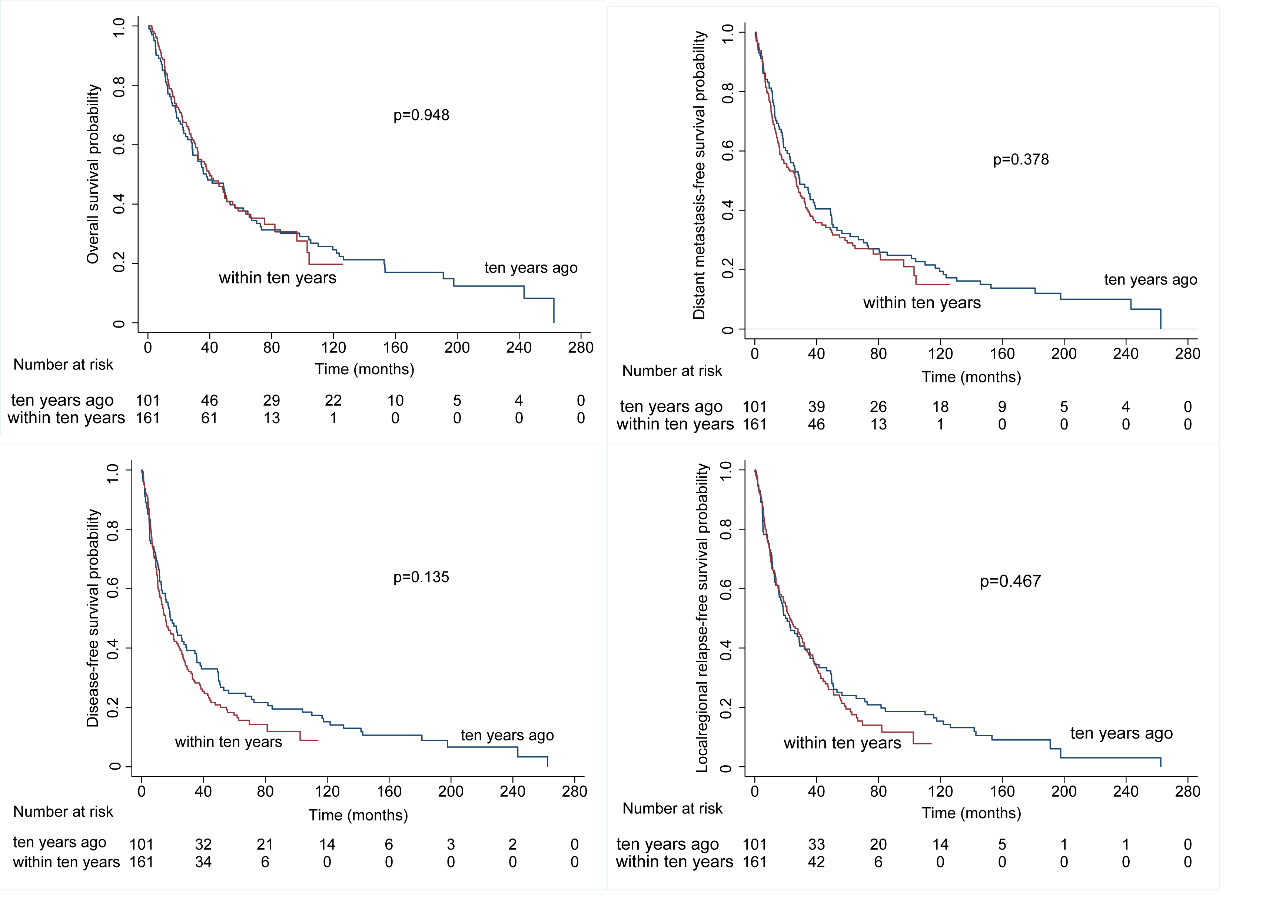
**

**Table S1.** Distribution of the use of immunotherapy/targeted therapy in patients receiving surgery, radiotherapy, and chemotherapy, respectively.

|  | **With Immunologic/targeted therapy** | **Without**  **Immunologic/targeted therapy** | ***P* value** |
| --- | --- | --- | --- |
| **Surgery** |  |  | 0.378 |
| Yes | 27 (39.1%) | 79 (40.9%) |  |
| No | 42 (60.9%) | 114 (59.1%) |  |
| **Radiotherapy** |  |  | 0.793 |
| Yes | 27 (39.1%) | 79 (40.9%) |  |
| No | 42 (60.9%) | 114 (59.1%) |  |
| **Chemotherapy** |  |  | <0.001 |
| Yes | 62 (89.9%) | 87(45.1%) |  |
| No | 7 (10.1%) | 106 (54.9%) |  |

**Table S2. Other studies evaluating the effect of multiple treatment in MMHN**

| Author | Sample size | Nature of the study | Country | Inclusion period | Groups | Overall survival (n-year results) | HR (95%CI) p value |
| --- | --- | --- | --- | --- | --- | --- | --- |
| Matthew Q^1^. | 1368 | National cancer database | USA | 2004-2012 | Surgery vs radiotherapy vs surgery +radiotherapy | 31% vs 8.1% vs 26.9% (5 year results) | 1 (reference) vs 2.49 (1.96-3.17) vs 1.06 (0.92-1.22); p<0.001 |
| Shiran Sun^2^. | 161 | Single center retrospective | China | 1981-2015 | Surgery vs surgery combined with  radiotherapy vs  Radiotherapy | 50.0% vs  43.1% vs 28.1% (5 year results) | Not reported |
| Enni Chen^3^ | 808 | National cancer database | China | 1975-2016 | Surgery vs radiotherapy vs surgery +radiotherapy | 20.6%vs10.9%vs27.2%   1. year results) | 1 (reference) vs 1.449 (1.010–1.742) vs 0.892 (0.753–1.056); p=0.006, 0.183 |

Reference

1. Schmidt MQ, David J, Yoshida EJ, et al. Predictors of survival in head and neck mucosal melanoma. Oral Oncol 2017;73:36-42.

2. Sun S, Huang X, Gao L, et al. Long-term treatment outcomes and prognosis of mucosal melanoma of the head and neck: 161 cases from a single institution. Oral Oncol 2017;74:115-22.

3. Chen E, Wu J, Liu Z, et al. Prognostic values of treatment modalities on head and neck mucosal melanomas in elderly patients: a population-based analysis. Ann Transl Med 2021;9:391.

**Dose and target delineation.**

The median radiotherapy dose was 58 Gy (range 30-70 Gy). Gross tumor volume (GTV) included the primary tumor and the enlarged lymph nodes, and GTV was expanded by 5-10 mm to generate the clinical tumor volume (CTV). For postoperative adjuvant radiotherapy, the CTV included the entire anatomical site where the tumors were located, and was expanded by 3-5 mm to generate the planning target volume (PTV). Cervical high-risk areas (including the number of metastatic lymph nodes ≥ 2, diameter ≥ 3 cm, extranodal lymph node invasion, and local recurrence after lymphatic dissection) were encompassed by the CTV.
